# Gerbode defect presenting as an atypical tricuspid regurgitation jet: recognizing the unrecognized

**DOI:** 10.1186/s44348-026-00105-5

**Published:** 2026-11-09

**Authors:** Moojun Kim, Geunhee Park, Joongmin Kim, Hyeongsoo Kim, Ji-Yong Jang, Hancheol Lee, Jong-Kwan Park, Seungjin Oh, Se-Jung Yoon

**Affiliations:** Division of Cardiology, National Health Insurance Service Ilsan Hospital, Goyang, Republic of Korea

**Keywords:** Congenital heart disease, Echocardiography, Intracardiac shunt, Tricuspid regurgitation, Computed tomography

A 52-year-old female patient with no significant medical or surgical history was referred to our institution due to cardiomegaly detected on a routine health screening. She was asymptomatic, and her initial electrocardiogram and chest x-ray showed unremarkable findings. Transthoracic echocardiography (TTE) revealed preserved left ventricular systolic function but demonstrated an enlarged right atrium (RA) and an unusual, strong tricuspid regurgitation (TR) jet (Fig. 1A, B, Video S1). Although the calculated right ventricular systolic pressure (RVSP) suggested severe pulmonary hypertension of 98 mmHg, there was no evidence of right ventricular (RV) enlargement or hypertrophy despite the presence of RA enlargement (Fig. 1C). Further evaluation of color Doppler raised suspicion for a ventricular septal defect (VSD); however, the left ventricular (LV) size, RV outflow tract velocity–time integral (14 cm), and Qp/Qs ratio (0.9) were all within normal limits (Fig. 1D, Video S2).

**Fig. 1:**
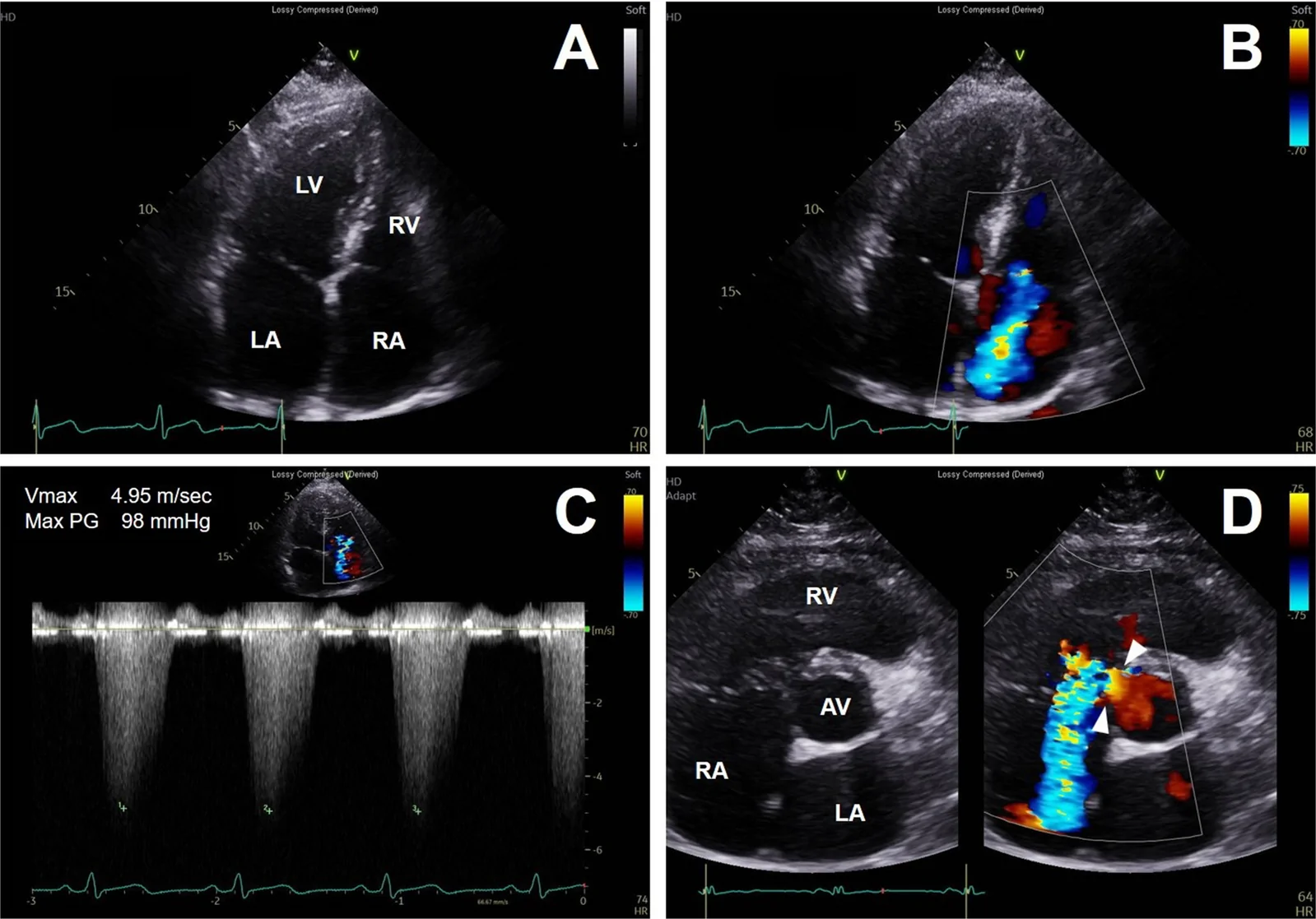
Apical four-chamber views and parasternal short-axis views. **A** Apical four-chamber view demonstrating dilated right atrium (RA) and a normal-sized right ventricle (RV). **B** Apical four-chamber view demonstrating prominent tricuspid regurgitation (TR) jet in RA. **C** Severe pulmonary hypertension (estimated pulmonary artery systolic pressure, 98 mmHg across the strong TR jet). **D** Abnormal shunt flow across the perimembranous septum in the parasternal short-axis view, suspicious for ventricular septal defect (arrowheads). AV, aortic valve; LA, left atrium; LV, left ventricle

Repeat TTE and subsequent transesophageal echocardiography revealed an atypical shunt flow communicating from the LV to the RA. The color jet after passing through the shunt impinged on the irregularly thickened tricuspid valve and subsequently deviated toward the RA (Fig. 2, Videos S3, S4). Additionally, another true mild TR flow indicating a normal RVSP of 20 mmHg was identified at the center of the tricuspid valve in the apical four-chamber view, which was distinct from the previously noted stroke jet flow. (Fig. 3, Video S5). Coronary computed tomography (CT) demonstrated an enlarged RA and a LV-to-RA shunt, which were consistent with a direct type Gerbode defect (Fig. 4). The patient had no prior history of cardiac surgery, catheter-based intervention, trauma or myocardial infarction or infective endocarditis.

**Fig. 2:**
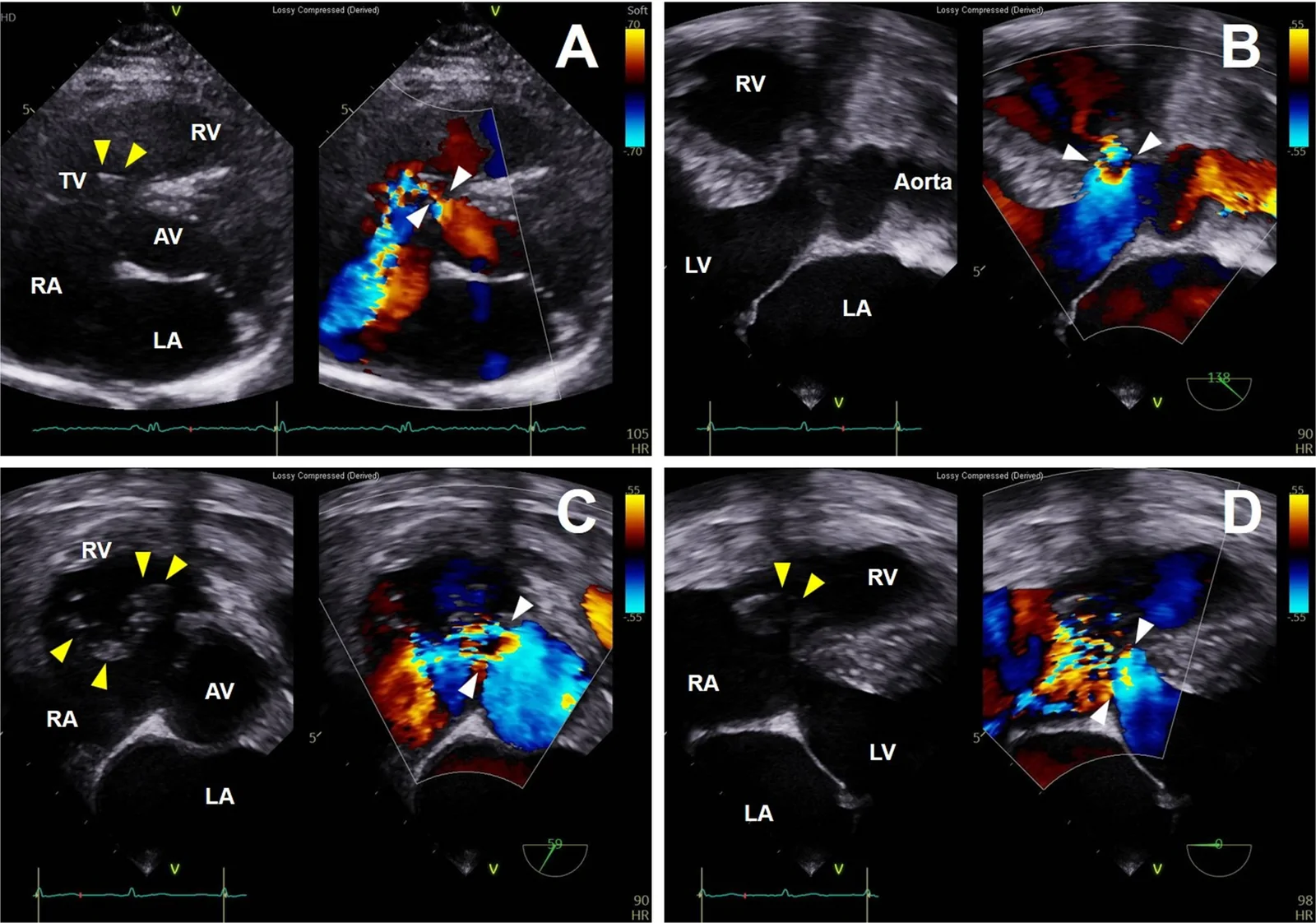
Modified parasternal short-axis views and transesophageal echocardiography (TEE). **A** A strong abnormal jet originating from the left ventricle (LV), impinging on the tricuspid valve (TV; white arrowheads, origin of shunt flow) and turning sharply toward the right atrium (RA; yellow arrowheads). **B** Strong jet flow across the perimembranous septum on TEE (138° view, arrowheads). **C**, **D** Strong LV-to-RA shunt flow across the perimembranous septum in TEE (59° and 0° views, yellow arrowheads, TV; white arrowheads, origin of shunt flow). AV, aortic valve; LA, left atrium; RV, right ventricle; TR, tricuspid regurgitation; VSD, ventricular septal defect

**Fig. 3:**
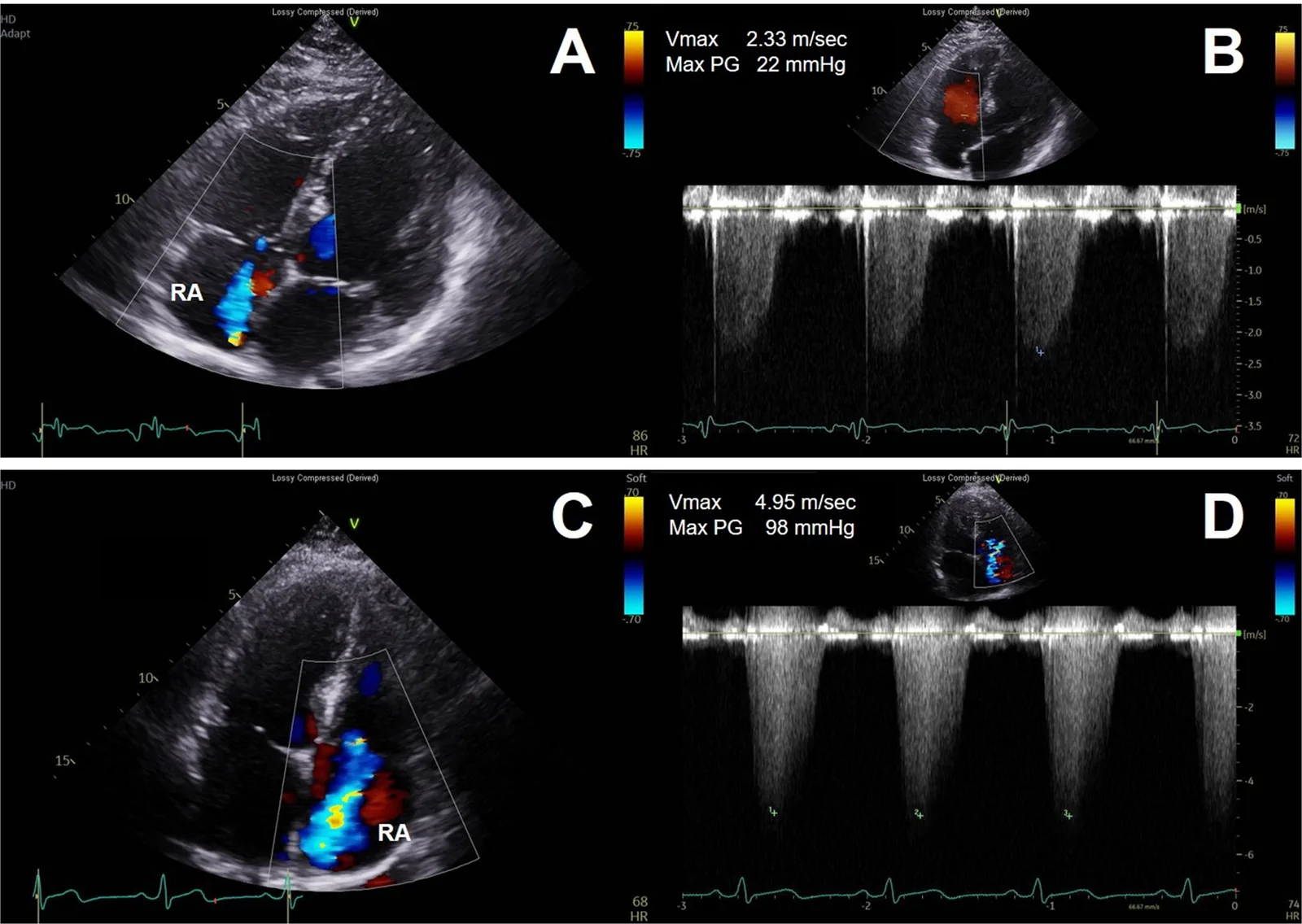
Apical four-chamber views. **A–D** Head-to-head comparison of a true tricuspid regurgitation jet and a left ventricle-to-right atrium (RA) shunt flow and each velocity

**Fig. 4:**
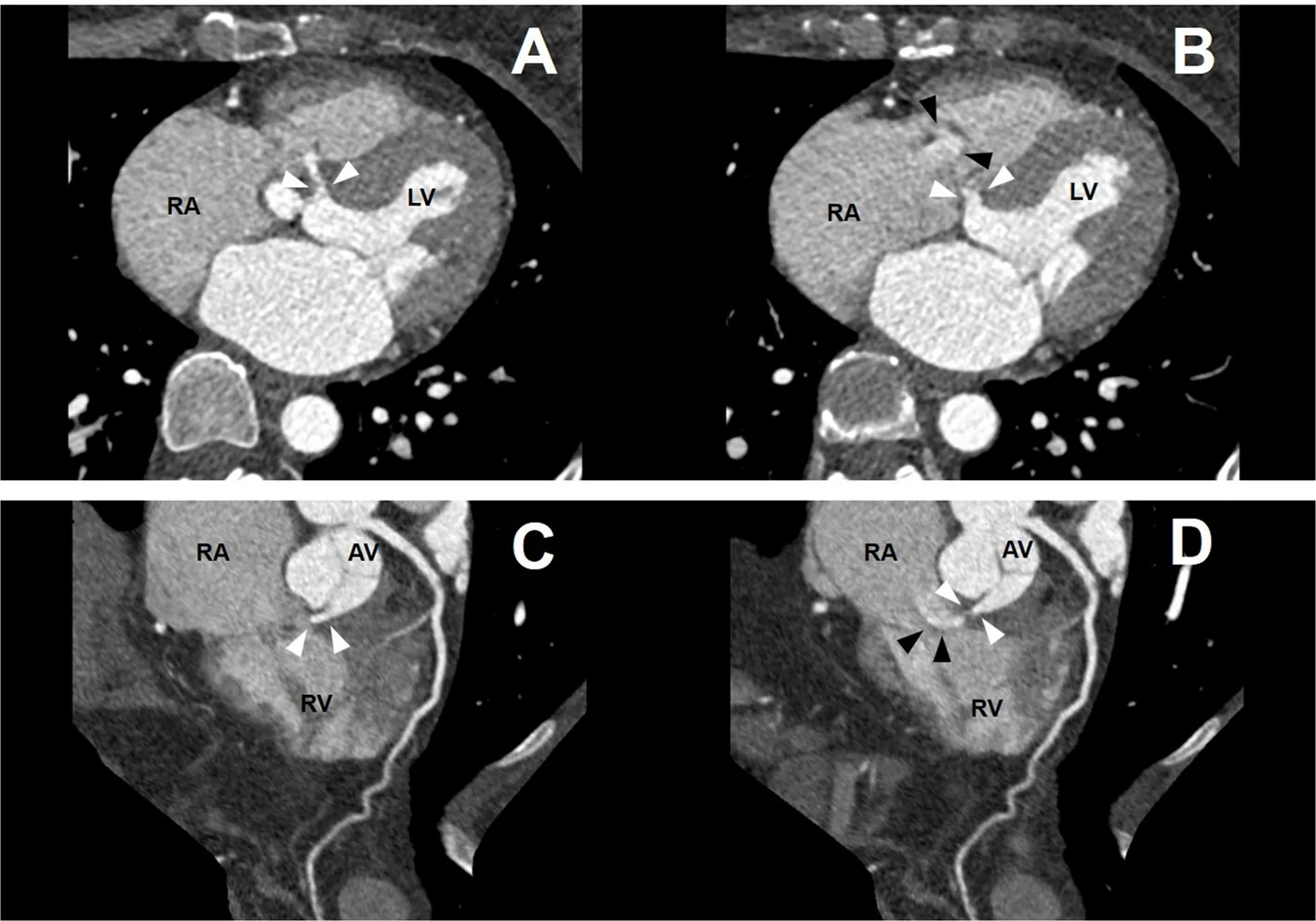
Cardiac computed tomography, axial and oblique sagittal views. **A–D** A small left ventricle (LV)-to-right atrium (RA) shunt flow through the perimembranous septum (white arrowheads) and a continuous shunt flow directed toward the RA (black arrowheads). AV, aortic valve; LV, left ventricle; RV, right ventricle

This case describes a congenital direct type Gerbode defect that mimicked a severe TR jet or VSD flow. It highlights that a Gerbode defect should be clinically considered when an unusually strong TR jet yields an unexpectedly high RVSP that does not correlate with the patient's clinical status, particularly when severe pulmonary hypertension is suggested in the absence of RV enlargement, RV hypertrophy or pulmonary arterial dilatation but in the presence of RA enlargement. In this case, the jet appeared to originate from the membranous septum of the LV rather than the tricuspid valve. Careful assessment of the jet origin and its pathway using color Doppler imaging is essential to avoid misdiagnosing the lesion as isolated TR or a VSD. Furthermore, referencing the origin of the shunt identified on CT can significantly facilitate a definitive diagnosis, preventing the misinterpretation of an LV-to-RA shunt as other condition with severe pulmonary hypertension.

Historically, Gerbode defects were recognized primarily as congenital anomalies resulting from embryological defects in the membranous septum, since its initial description in 1958 [1, 2].

Subsequent reports further classified Gerbode defects into two anatomical subtypes: direct type I (supravalvular) LV-to-RA shunts and indirect type II (infravalvular) LV-to-RV-to-RA shunts with cleft-shaped or perforation of tricuspid valve (TV), according to the location of the communication and morphology of TV [2, 3]. However, the definitions of type I and type II Gerbode defects are not entirely consistent across published reports [4]. Yuan [5] proposed a comprehensive classification incorporating both congenital and acquired forms of Gerbode defects in 2014. There were a few reports presenting that congenital Gerbode defects account for the majority of indirect infravalvular type [6, 7]. However, with the increasing frequency of invasive cardiac interventions and surgical procedures in the modern era, recent systematic reviews have revealed that acquired forms now predominate, accounting for approximately 73% of all reported cases [5, 8].

Gerbode defects are easily misidentified as atypical TR or VSD [9, 10]; therefore, precise evaluation using color Doppler echocardiography or CT is essential for a definitive diagnosis [11]. Here we report a rare case of an adult presentation of a silent, congenital direct type supravalvular Gerbode defect that was initially approached as atypical TR or a VSD.

## Supplementary Information

Additional file 1: Video S1. Transthoracic echocardiography demonstrating an unusual, prominent tricuspid regurgitation jet in apical four-chamber view.

Additional file 2: Video S2. Transthoracic echocardiography showing a severe tricuspid regurgitation jet and a suspected ventricular septal defect in parasternal short-axis view.

Additional file 3: Videos S3. An atypical shunt flow communicating from the left ventricle to the right atrium in modified parasternal short-axis view and transesophageal echocardiography.

Additional file 4: Videos S4. An atypical shunt flow communicating from the left ventricle to the right atrium in modified parasternal short-axis view and transesophageal echocardiography.

Additional file 5: Video S5. Another true mild tricuspid regurgitation flow at the center of the tricuspid valve in the apical four-chamber view.

## Data Availability

Data available on request. The data underlying this article will be shared on reasonable request to the corresponding author.

## Abbreviations

CT: Computed tomography
LV: Left ventricular
RA: Right atrium
RV: Right ventricular
RVSP: Right ventricular systolic pressure
TR: Tricuspid regurgitation
TTE: Transthoracic echocardiography
TV: Tricuspid valve
VSD: Ventricular septal defect
